# Investigating microstructural and mechanical properties evolution in biomedical AZ31 magnesium alloy under different casting conditions

**DOI:** 10.1038/s41598-025-28526-0

**Published:** 2025-12-29

**Authors:** Gunvanta Dhanuskar, Abhaykumar Kuthe, Dheeraj Bhiogade, Bhupesh Sarode, Ashutosh Bagde

**Affiliations:** 1 Department of Mechanical Engineering, Visvesvaraya National Institute of Technology, Nagpur, 440010 Maharashtra India; 2 Faculty of Engineering and Technology, Datta Meghe Institute of Higher Education & Research, Sawangi (M), Wardha, 442005 Maharashtra India; 3 Jawaharlal Nehru Medical College, Datta Meghe Institute of Higher Education and Research, Sawangi (M), Wardha, 442005 Maharashtra India

**Keywords:** Magnesium alloy, Metal casting, Microstructural analysis, Customized implant, Mechanical properties, Biomedical metallic materials., Engineering, Materials science

## Abstract

Magnesium alloy AZ31 is highly valued for aerospace components, medical implants, and prosthetics due to its exceptional mechanical properties. Its density and elastic modulus closely match those of cortical bone, making it a promising material for orthopaedic applications. Its biodegradability further enhances its potential by eliminating the need for secondary surgeries. However, casting magnesium alloy AZ31 poses challenges due to its high reactivity, leading to oxidation, porosity, and phase segregation, which affect mechanical properties and surface integrity. This study focuses on a method for developing a customized, patient-specific AZ31 magnesium alloy implant using casting as the manufacturing technique. The microstructure and mechanical properties of AZ31 are investigated with respect to different casting parameters, pouring temperatures, shielding techniques, and furnace types. SEM and EDS identified primary α-Mg and secondary β-Mg17Al12 phases, as well as increased oxide layer thickness and phase segregation. XRF and XRD were used to confirm shifts in alloy composition and the presence of phases such as MgO and Al-Mn intermetallic, which have been shown to effect on mechanical properties. This study aims to develop an effective method for casting AZ31 magnesium alloy implants, giving significant insights to minimize oxidation while improving the mechanical properties and surface integrity of AZ31 alloy castings.

**Supplementary Information:**

The online version contains supplementary material available at 10.1038/s41598-025-28526-0.

## Introduction

**Fig. 1:**
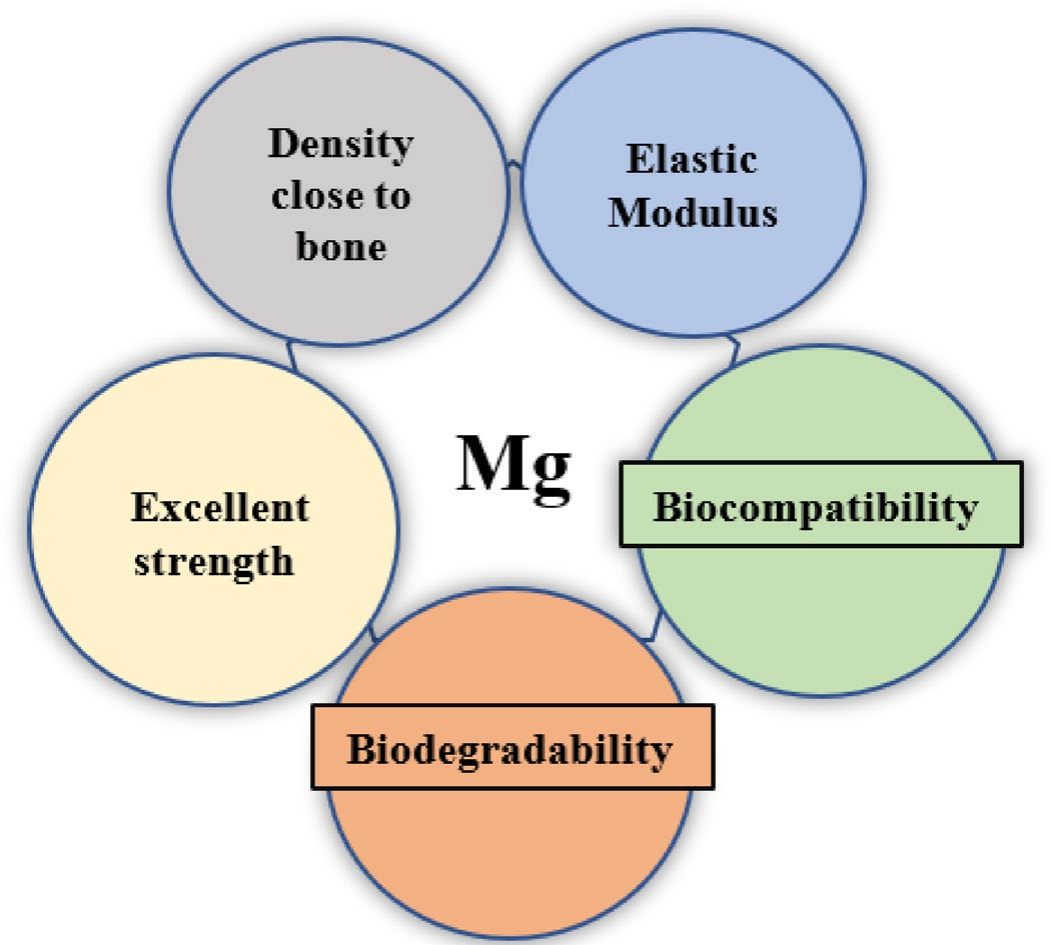
Biomedical-relevant properties of magnesium for implant design.

Magnesium alloys are extremely valued for their environmental compatibility^1^ and various applications in the automotive, aerospace, computer, communication, and biomedical^2^. AZ31 magnesium (Mg) alloy is prominent because of its excellent strength-to-weight ratio, machinability, and biocompatibility. These properties make it an attractive material for applications requiring reduced weight without the contribution of mechanical strength. Mg is suitable for biomedical applications due to its biocompatibility, biodegradability, and bioabsorbability^3^. Biodegradable metallic materials, particularly Mg and its alloys, have emerged as a promising material for biomedical implant applications owing to their unique combination of controlled degradability and favourable mechanical properties. Among metallic biomaterials, Mg exhibits a density of approximately 1.7 g/cm³, which is the closest to that of natural cortical bone (1.8–2.1 g/cm³), thereby minimizing stress shielding effects^4,5^. The principal advantages of Mg for Orthopedic applications are summarized in Fig. 1. One of the most remarkable advantages of these materials lies in their ability to eliminate the need for a secondary surgery to remove the implant after healing is complete. Unlike conventional metallic implants made from stainless steel, cobalt-chromium alloys, or titanium alloys, which remain permanently in the body or require surgical removal, Mg implants gradually degrade over time in vivo through natural corrosion processes. However, despite its advantages, Mg has a high oxidation rate during casting, posing a significant challenge, compromising the mechanical properties and surface integrity of the alloy. This emphasizes the need for improved casting methods and effective shielding strategies to minimize oxidation and enhance the performance of Mg-based implants. Resolving these issues is crucial to maximising Mg alloy potential for creating biodegradable implants.

Among the conventional processes for making metallic bone implants, casting is considered the cost-effective manufacturing process. Its simplicity in process allows for further enhancements of material properties through thermal processes. Additionally, the casting process offers greater flexibility in regulating the concentration of alloying elements, enabling precise control over the overall alloy composition. Alloying Mg further enhances its properties, broadening its range of applications. However, Mg alloys oxidize quickly during processing in high-temperature environments compared to many other metals. Because Mg has a high reactivity and strong affinity with oxygen, its oxidation film on the surface is loose and porous, providing insufficient protection against further oxidation. This susceptibility can lead to severe oxidation or even combustion at high temperatures. Therefore, it is important to implement measures to prevent oxidation and combustion during the casting operations of Mg alloy^6,7^. The literature indicates that a significant number of Mg-based alloys are predominantly processed through the liquid phase. The liquid phase processing can be classified into sand casting, squeeze casting, stir casting, and high-pressure gravity casting are commonly employed for processing these alloys^8^. However, because Mg has a high affinity for oxygen, achieving a defect-free cast product remains challenging. The formation of oxides and intermetallic phases during casting can lead to mechanical defects, such as porosity and brittleness^9,10^. Exploration into enhancing the oxidation resistance of Mg alloys at high temperatures is crucial for expanding their applications. Fortunately, recent advancements in research have led to the development of new high-temperature Mg alloys that exhibit significantly improved corrosion and oxidation resistance. However, challenges such as oxidation and ignition during Mg alloys’ melting and casting processes persist. Numerous methods have been studied to mitigate or prevent the oxidation of molten Mg and its alloys in air, including flux protection, fluxless protection process, and the addition of alloying elements. From these, fluxless process using gas (Ar, Air, N_2_, He, CO_2_, Cl_2_, SF_6_, BF_3_, HC, SO_2_, and HFC) shielding is currently considered the most effective technique for protecting the melt^11^. The SF_6_ gas mixture method is recognized as highly effective for protecting molten Mg and its alloys due to its ability to create a stable and cohesive film on the melt surface^12,13^. However, SF_6_ has been found to contribute significantly to the greenhouse effect, which has led to restrictions or bans on its use in some countries. Consequently, there is a vital need to develop alternative methods that are both environmentally sustainable and less toxic, addressing the dual concerns of environmental impact and safety. Expensive SF_6_ in air protects molten Mg well from oxidation. That air is necessary to build a protective film on the melt surface. Inert gases like nitrogen and argon will not oxidize the metal, but the metal will evaporate since no film forms on the metal. An inexpensive CO_2_ can contain at least 20% air and be protective. Difficulties employing CO_2_ are that the metal surface gets discoloured, which is at least a superficial problem, and that C may be introduced into the metal, which may cause corrosion problems^13^. Argon is the cheapest and most effective for shielding molten Mg among all the gases. It is inert, has no environmental impact, and does not contribute to global warming or ozone depletion. In contrast, SF_6_ is a potent greenhouse gas with a global warming potential 23,500 times higher than CO_2_, which remains in the climate system for thousands of years^14,15^. While argon is slightly less effective in preventing oxidation, it is a more environmentally friendly and safer alternative. Argon is highly thermally stable. It remains inactive and does not undergo significant chemical reactions or dissolve at typical operating temperatures^13^. Understanding the impact of alloying elements on the oxidation behaviour of Mg and the composition of oxidation films formed in different atmospheres is crucial for enhancing the protection of Mg alloys. Research focusing on the oxidation characteristics of Mg alloys under various atmospheric conditions is essential to develop more effective methods for safeguarding these alloys.

The mechanical properties of biodegradable materials are influenced by several factors, which can be broadly categorized into internal and external factors. Internal factors pertain to the material itself, including aspects such as microstructure, strain, and surface roughness. On the other hand, external factors involve environmental influences, such as blood flow, body temperature, and pH value, which play a critical role in biomedical applications^16^. The internal factors are significantly impacted by the oxidation of Mg alloys during the casting process. Oxidation can significantly alter the microstructure by forming loose and porous oxide layers, which degrade surface integrity and mechanical performance. This could lead to a decrease in tensile strength, an increase in brittleness, and a higher probability of mechanical defects such as porosity. Additionally, the formation of undesirable phases during casting exacerbates these issues by contributing to inhomogeneous microstructures and compromising overall material reliability. Erstwhile studies have extensively explored the mechanical and structural properties of AZ31 Mg alloy under different manufacturing processes, focusing on rolling and extrusion. Research has demonstrated that the rolling process improves grain refinement and mechanical properties by reducing porosity and enhancing the formation of a homogeneous α-Mg phase^17,18^. However, casting processes, particularly those conducted at high temperatures, are more prone to producing inhomogeneous microstructures and undesirable phases, such as Mg₁₇Al₁₂ and MgO. Protective atmospheres, such as argon gas shielding, have been studied to mitigate oxidation during casting, with some success in reducing the formation of oxidized layers. However, the impact of sand-casting conditions in the production of biodegradable customized implants, such as temperature, furnace type, and the presence or absence of argon gas shielding, on the alloy’s microstructure and mechanical properties remains insufficiently explored. While substantial research has been dedicated to enhancing AZ31 alloy’s properties through rolling and extrusion, casting processes need further investigation. A deeper understanding is required of how the casting conditions affect oxidation behaviour, phase stability, and porosity in AZ31 alloy.

In this study stir sand casting method was used as the customized implant manufacturing technique and its characterization. The microstructure and mechanical properties of AZ31 alloy casting were examined under various casting parameters, including pouring temperatures, shielding techniques (investigating the behaviour of Mg AZ31 alloy under Argon protective and unprotected atmospheres at 550 °C and 630 °C), and different furnace conditions. These casting conditions influence the oxidation behaviour, phase stability, and porosity of AZ31 alloy. Thermodynamic models, such as Gibbs free energy calculations, could provide valuable insights into phase formation during casting by employing JMatPro (Version 7.0.0) software to predict the alloy’s melting and ignition temperatures. SEM (Scanning Electron Microscopy) and EDS (Energy Dispersive X-ray Spectroscopy) identified primary α-Mg and secondary β-Mg17Al12 phases, while also investigating increased oxide layer thickness and phase segregation in the cast sample. XRF (X-ray fluorescence) and XRD (X-ray diffraction) were used to confirm shifts in alloy composition and the presence of phases like MgO and Al-Mn intermetallic, which further effect on mechanical properties. This study helps to develop a process for Mg alloy implant metal casting, also providing insights into the casting process to eliminate oxidation and improve the mechanical properties and surface integrity of Mg AZ31 alloy casting.

## Materials and methods

**Fig. 2:**
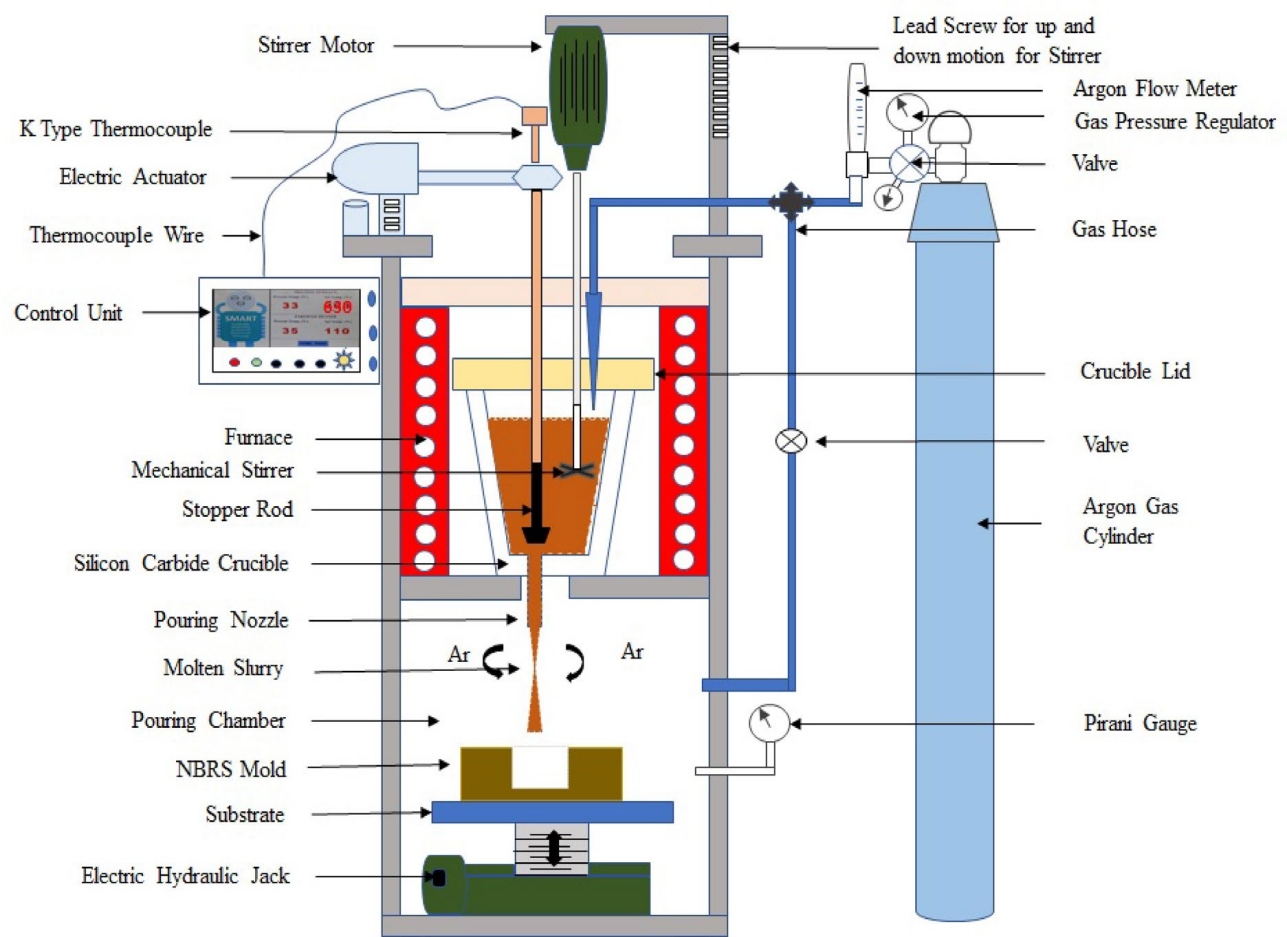
Schematic representation of a controlled casting SMART foundry setup.

The schematic diagram of the casting SMART (Sustainable Metal Casting using Advanced Research & Technology) Foundry experimental setup is shown in Fig. 2, detailing the AZ31 Mg alloy casting process to represent the key components and procedures employed during the experiments. For the experiment, a commercial AZ31 Mg alloy roll sheet, procured as raw material from Exclusive Magnesium, Hyderabad, India, was used. The chemical composition (wt%) of AZ31 Mg alloy is shown in Table 1, including the standard values specified by ASTM B90^19–21^ and the XRF data for both the raw material and the cast samples. The standard composition and associated material properties were obtained from the MatWeb materials database^20^. In each experiment, specimens were prepared from the rolled sheet with dimensions of 40 mm in length, 15 mm in width, and 2 mm in thickness. Approximately 100 g of AZ31 alloy was melted in a silicon carbide crucible with a top outer diameter of 92 mm, bottom diameter of 65 mm, and height of 100 mm.

**Table 1:** Standard and XRF-determined chemical composition of AZ31 Mg alloy (wt%).

Element (wt%)	Standard AZ31 (ASTM B90) [19–21]	Rolled Sheet AZ31 (XRF – As Raw Material)	Cast AZ31 (XRF – After Casting)
Al	2.50–3.50	3.426	3.135
Zn	0.60–1.40	1.062	0.860
Mn	≥ 0.20	0.261	0.296
Si	≤ 0.10	0.265	0.270
Fe	≤ 0.0050	0.002	0.008
Cl	-	0.112	0.215
S	-	0.118	0.006
Other	0.30	0.852	0.35
Mg	Balance	Balance	Balance

**Fig. 3:**
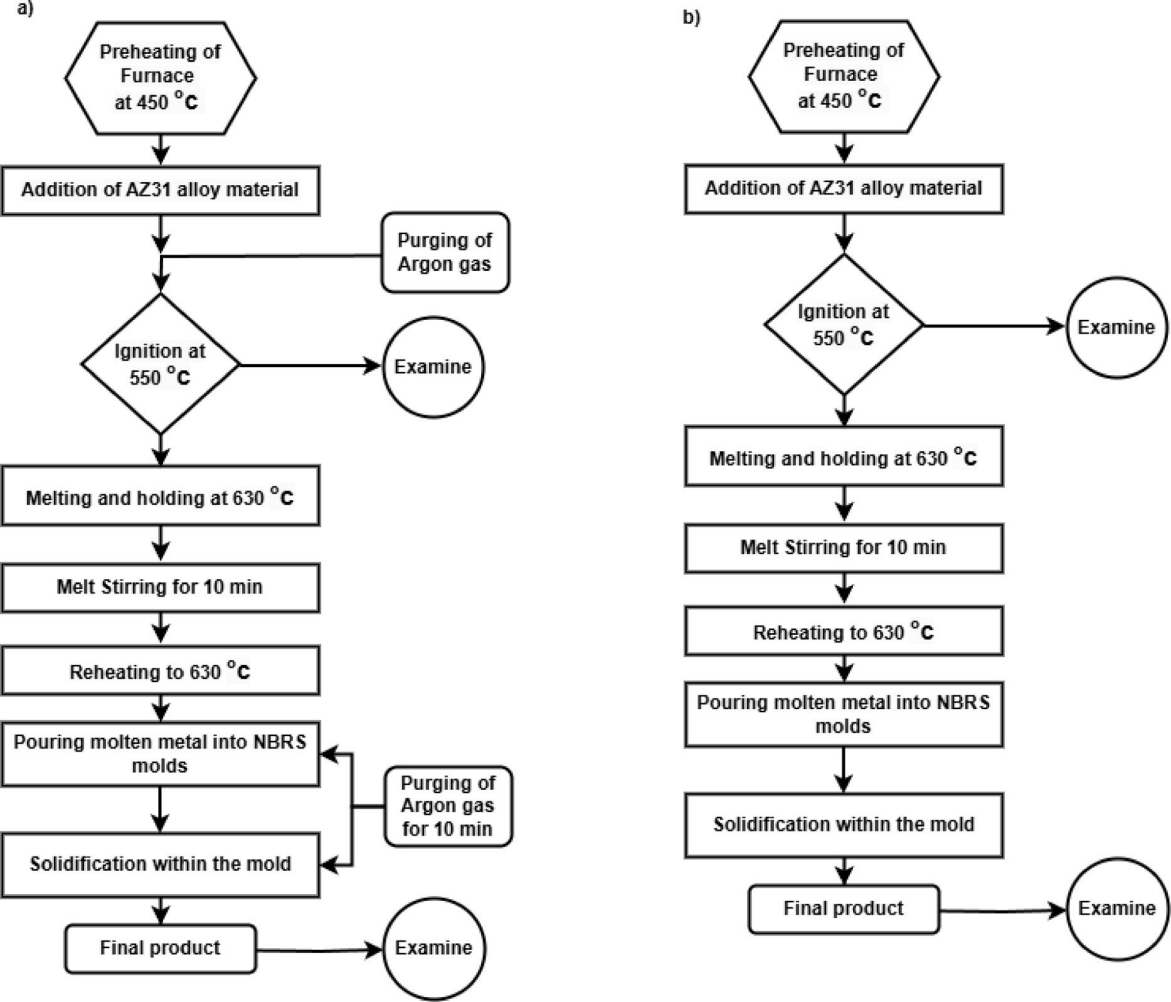
Flow process charts for AZ31 Alloy casting (**a**) with argon gas shielding and (**b**) without argon gas shielding.

A total of eight experiments were conducted, with each experiment repeated three times. The experimental conditions, including furnace processing parameters at different temperatures and casting environments, are summarized in Table 2. The flow process charts for AZ31 alloy casting with and without Argon gas shielding are shown in Fig. 3, illustrating the procedural variations in the casting process. This experiment used a muffle furnace and an electrical resistance furnace for the melting of metal. A muffle furnace is a high-temperature heating system with a closed refractory-lined chamber that keeps the material isolated from direct contact with heating elements. This design creates a controlled atmosphere, which prevents contamination from combustion by-products during high-temperature processing^22,23^. In contrast, an open-chamber resistance furnace uses indirect resistance heating, where the current flows through separate heating elements, which then transfer heat to the workpiece^24^.

**Table 2:** Experimental parameters for furnace processing across temperatures and casting environment types.

Exp. *N*.	Furnace Type	Temperature	Casting Environment
1	Closed Chamber (Muffle) Furnace	550 °C	Unprotected
2	Open Chamber (Resistance) Furnace	550 °C	Unprotected
3	Closed Chamber (Muffle) Furnace	630 °C	Unprotected
4	Open Chamber (Resistance) Furnace	630 °C	Unprotected
5	Closed Chamber (Muffle) Furnace	550 °C	Argon Gas Protected
6	Open Chamber (Resistance) Furnace	550 °C	Argon Gas Protected
7	Closed Chamber (Muffle) Furnace	630 °C	Argon Gas Protected
8	Open Chamber (Resistance) Furnace	630 °C	Argon Gas Protected

**Fig. 4:**
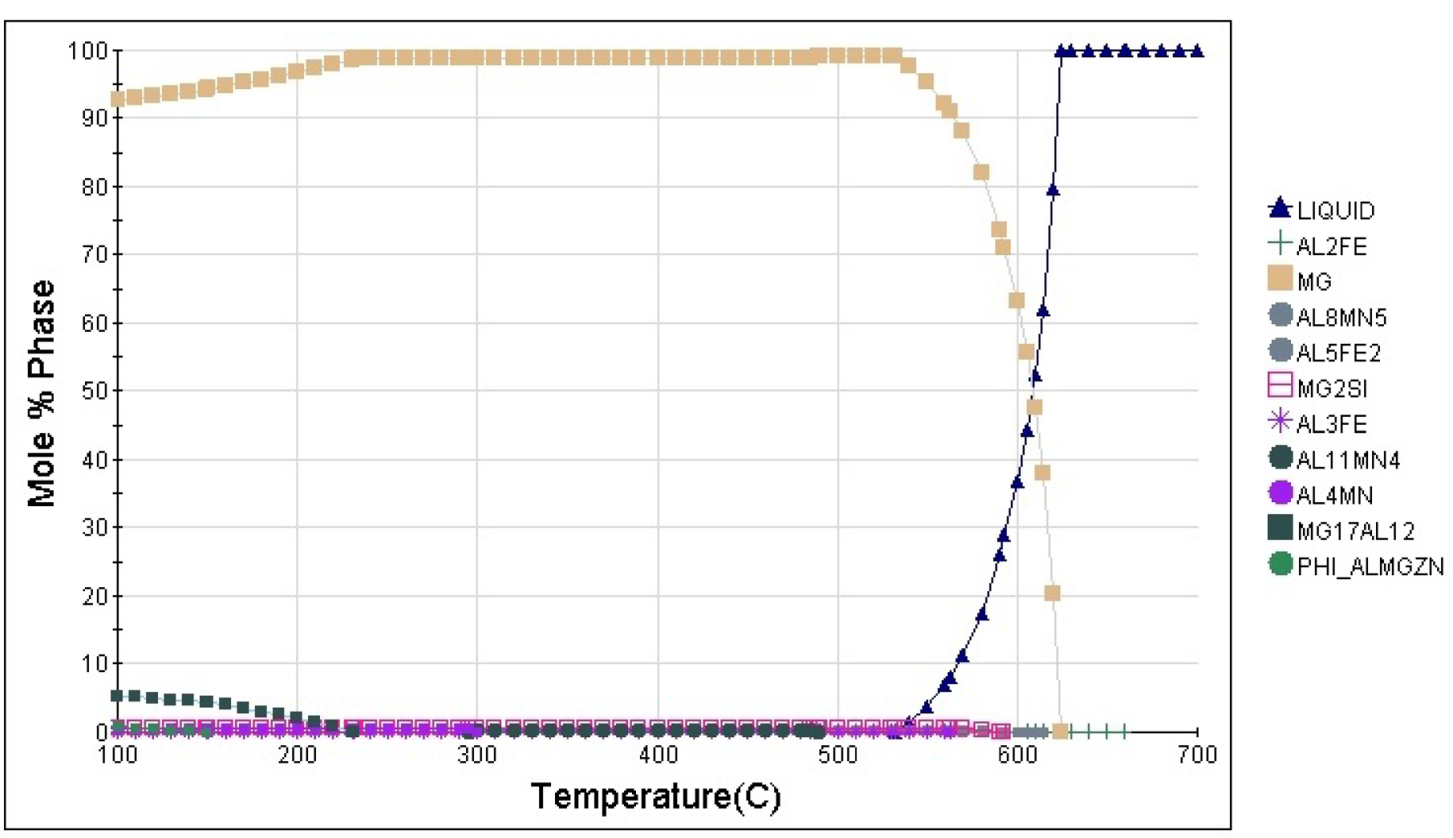
Phase identification using JMatPro (Version 7.0.0) software.

Based on data from the literature and the JMatPro (Version 7.0.0, by Sente Software Ltd, Guildford, UK) software^25^ (which computes various material properties for alloys), temperatures were chosen for the experiment^26^. Figure 4 shows the weight% of different phases present in the material in relation to temperature. The melting temperature, or the point at which a solid substance becomes a liquid, is around 630 °C. This is the temperature at which the transition from the solid to the liquid phase is complete, as shown in the figure. About 550 °C is the minimum ignition temperature at which a substance will ignite and continue to burn when exposed to an oxidizing agent. This is where an exothermic reaction, caused by ignition, releases energy^27,28^.

In an electrical resistance furnace and a muffle furnace, the raw material was heated to two temperatures, 550 °C and 630 °C, with a ± 2 °C accuracy, for 60 and 90 min, respectively. The temperature is measured using a K-type thermocouple connected to a control unit, ensuring precise thermal regulation. In an unprotected environment, the heating rates required to achieve the predetermined temperatures were 9 °C/min and 7 °C/min, respectively. It takes approximately ± 10 min longer to reach these temperatures in a protected environment, resulting in a variation in the heating rate. The crucible was not sealed in the muffle furnace during the heating process. In contrast, in the resistance furnace, the crucible was encased with a ceramic plate to mitigate the environmental impact on the material and to diminish the possibility of complete oxidation and probable fire^29,30^. In this experiment, the molten metal is poured into no-bake resin sand (NBRS) moulds placed on a substrate using an electric hydraulic jack for controlled pouring rate, and the molten metal is allowed to solidify within these moulds under the specified experimental conditions. Since the process involved controlled melting followed by mould solidification, it represented a casting process rather than a heat treatment of the rolled sheet. The furnaces in the SMART Foundry setup functioned as melting units to obtain cast specimens under different temperature and shielding conditions. The experiment was conducted in two types of environments, one with argon gas shielding and the other without. Argon is an odourless and colourless non-combustible gas that is heavier than air. It was supplied through a flow meter and pressure regulator to minimize oxidation of the molten alloy^13^. In the argon gas shielding environment, once the temperature reached about 450 °C, argon was injected into the chamber at a flow rate of 20 l/min and a pressure of 1.5 kg/cm^2^. The flow of gas was maintained constant until the required experimental temperature was reached. After the molten metal was poured into the mould, the argon flow in the pouring chamber was maintained for an additional 10 min during the solidification process. In the absence of argon gas shielding, entire processes, including heating, melting, pouring, and solidification, were conducted without the use of any protective atmosphere. This process, as demonstrated in Fig. 3**(b)**, emphasised the procedural flow for AZ31 alloy casting without argon gas shielding, comparing it with the shielded environment shown in Fig. 3**(a).**

This manufacturing process significantly affects the microstructure and, consequently, the mechanical and physical properties of the alloy. Following the experiment, various techniques for analysis were employed to examine the characteristics of the oxides that formed on the AZ31 alloy. A combination of XRD, XRF, and SEM with EDS was utilized to comprehensively analyse the structural and compositional changes induced by the casting process in the successfully cast AZ31 alloy sample. For microstructural analysis, specimens were ground sequentially with SiC papers up to 2000 grit, polished with diamond paste (1 μm and 0.5 μm), and subsequently etched using an acetic–picric solution (4.2 g picric acid, 10 ml acetic acid, 10 ml water, and 70 ml ethanol). The samples were immersed in the etchant solution for 1 s and then washed with distilled water and ethanol^31^. These procedures ensured a smooth, oxide-free surface suitable for optical microscopy and SEM analysis. An extensive analysis of the oxidation process, encompassing the surface morphology, elemental composition, and molecular structure of the cast products, was made possible by this multidimensional technique. This study compares the hardness, density, and porosity results of a raw material and a successfully cast sample to understand how processing methods affect material mechanical properties. This information is important for enhancing the performance of cast AZ31 alloys in medical applications, especially where thermal stability, mechanical strength, and oxidation resistance are vital.

## Results and discussion

### Photomacroscopic observations

**Fig. 5:**
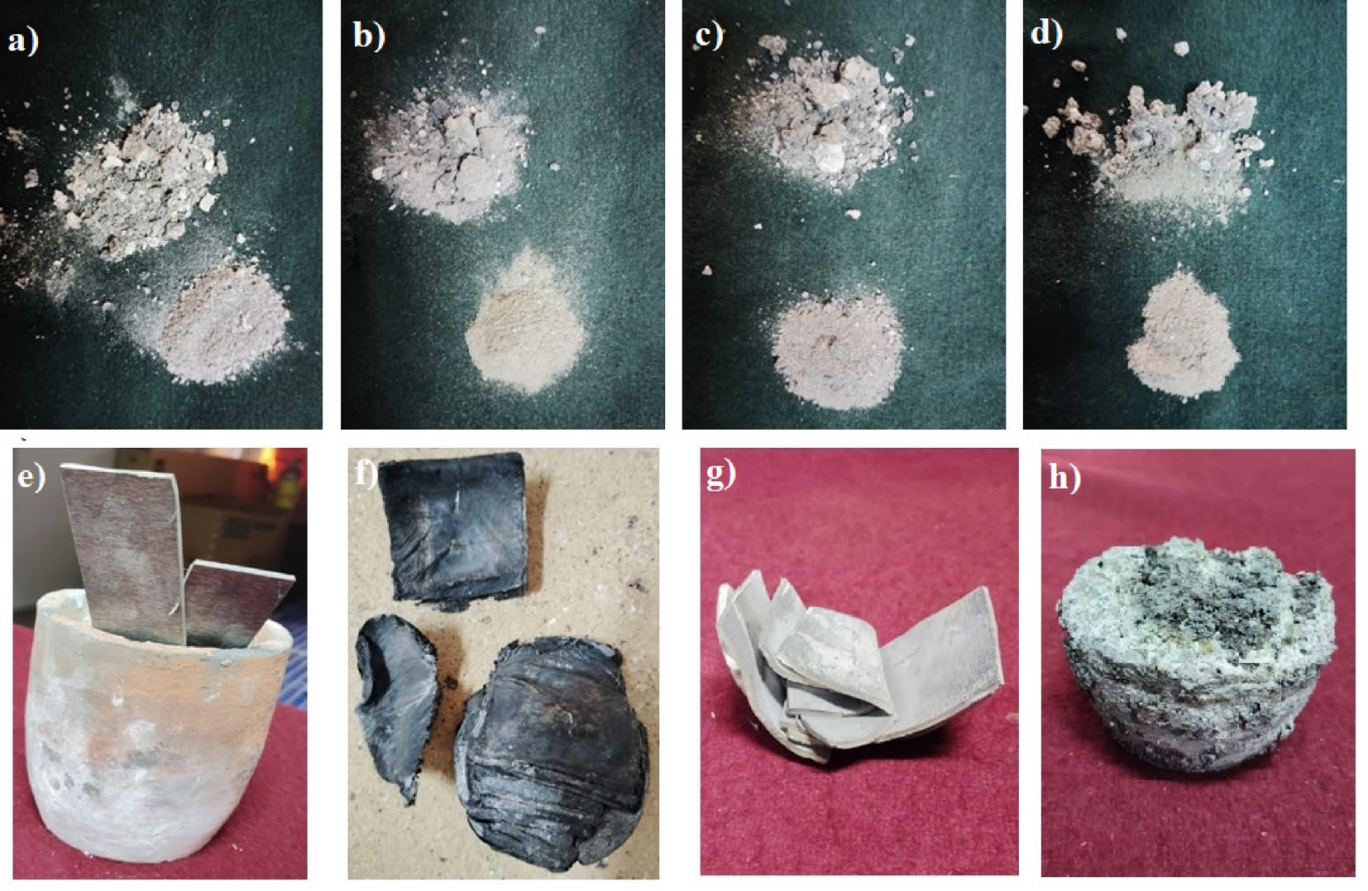
Photomacrograph of the AZ31 Mg alloy heated at different condition (Exp-1 to Exp-8); (**a**) Exp-1: 550 °C without argon gas shielding in Muffle furnace, (**b**) Exp-2: 550 °C without argon gas shielding in Resistance furnace, (c) Exp-3: 630 °C without argon gas shielding in Muffle furnace, (**d**) Exp-4: 630 °C without argon gas shielding in Resistance furnace, (**e**) Exp-5: 550 °C with argon gas shielding in Muffle furnace, (**f**) Exp-6: 550 °C with argon gas shielding in Resistance furnace, (**g**) Exp-7: 630 °C with argon gas shielding in Muffle furnace, (**h**) Exp-8: 630 °C with argon gas shielding in Resistance furnace.

Figure 5 displays photomacrographs of AZ31 Mg alloy cast under various conditions described in Table 2, following the casting procedure outlined in Fig. 3. In environments without argon gas shielding, AZ31 material was placed in a preheated crucible at 450 °C and then heated to 550 °C and 630 °C. In the muffle furnace, a closed chamber, the material had limited contact with air, whereas in the resistance furnace, an open chamber, the material was in constant contact with air. At both 550 °C and 630 °C in the muffle furnace, the metal gradually ignited, burned completely, and sublimed. However, in subsequent trials using a resistance furnace at the same casting conditions, the material experienced more severe oxidation and intense ignition due to oxygen exposure, resulting in the complete sublimation of the residual material in the crucible. Figure 5**(a)**,** (b)**,** (c)**,** and (d)** illustrate these phenomena attributed to the oxidation observed after the liquid Mg sublimed. Due to the sublimation of Mg at elevated temperatures, only a minimal amount remained^32,33^. In the subsequent four experiments, argon gas shielding was provided. When the materials were placed in a preheated crucible at 450 °C in the muffle furnace and resistance furnace, and argon gas was purged into the heating chamber, it was observed that when heated to 550 °C and 630 °C, the heating rate decreased compared to without argon shielding environment. The argon gas shielding required more time to reach the ignition and melting temperatures. Another observation, when heating in the muffle furnace with argon shielding in Fig. 5**(e)**, shows that at 550 °C, the metal did not melt, and in Fig. 5**(g)** shows that at 630 °C, the temperature was sufficient only to fuse the plates, reaching the calcination temperature. The material fused at this temperature but did not fully melt because the argon gas shielding prevented it from reaching the melting point, as the heating rate was affected by the shielding. As a result, oxidation granules appeared on the surface of the material as a greyish powder. In the resistance furnace, at 550 °C, the upper part of the material melted but not properly, appearing black with more oxidation observed, as shown in Fig. 5**(f)**. At 630 °C, the metal melted adequately, forming a homogenized liquid bed. After the molten metal solidified in the NBRS mould, the upper granules of the metal were severely oxidized and vaporized, while less oxidation was observed on the bottom part of the material compared to the upper part, as shown in Fig. 5**(h).**

The above highlights the significant impact of heating temperature, duration, and casting environment on granule appearance and oxidation behaviour, particularly under argon shielding. Additionally, the presence of argon gas significantly affects the heating temperature and heating rate, because argon has lower thermal conductivity compared to air. As a result, when argon gas is used in a furnace, it can slow down the rate of heat transfer to the material. This leads to a slower heating rate, as the gas acts as an insulating layer around the material, reducing the efficiency of heat exchange^34^. As a result, the material did not achieve complete melting in the closed chamber with argon gas protection at the selected melting temperature, instead attaining only a semi-solid state close to its calcination temperature. In contrast, in the open chamber with argon gas shielding, successful casting occurred at 630 °C with minimal oxidation, but the mechanical properties deteriorated compared to the raw material. This decline in properties could be attributed to microstructural changes during the casting process despite the reduced oxidation. Grain coarsening and phase changes likely contributed to decreased mechanical properties. The grey hue on the granule surface indicates uniform oxide formation. At the same time, black patches are likely due to carbon deposits from Mg reacting with carbon dioxide in the air, as described in Eqs. (1–2)^30,35^.

1 $$Mg (L) +{1}/{2}\:O_{2} (G) \rightarrow\: MgO (S)$$

2 $$Mg(L)+1/2\: CO_{2} (G) \rightarrow\: MgO (S)+ 1/2\:C(S)$$

It was also observed that in Exp-6 and Exp-7, the materials loosely arranged themselves, conforming to the shape of the crucible. A small amount of liquid Mg formed within the magnesium oxide (MgO) during the alloy’s oxidation, which acted as a binder, leading to the agglomeration of non-melted meta^30^, as illustrated in Fig. 5**(f) and (g**). From Fig. 5**(a)**,** (b)**,** (c)**,** and (d)**, it is evident that no signs of crucible adhesion occurred when heated at 550 °C and 630 °C without argon gas shielding in both furnaces. However, after heating at these temperatures with argon shielding in both furnaces, slight traces of crucible adhesion were observed on the material surfaces, as shown in Fig. 5**(f)**,** (g)**,** and (h)**. This minimal adhesion suggests a reaction between the crucible and the metal. The excellent thermal conductivity of SiC crucibles ensures uniform temperature distribution during the melting process, likely contributing to the observed minimal adhesion. Additionally, their chemical stability and resistance to corrosion play a crucial role in preventing contamination of the AZ31 Mg alloy during melting.

**Fig. 6:**
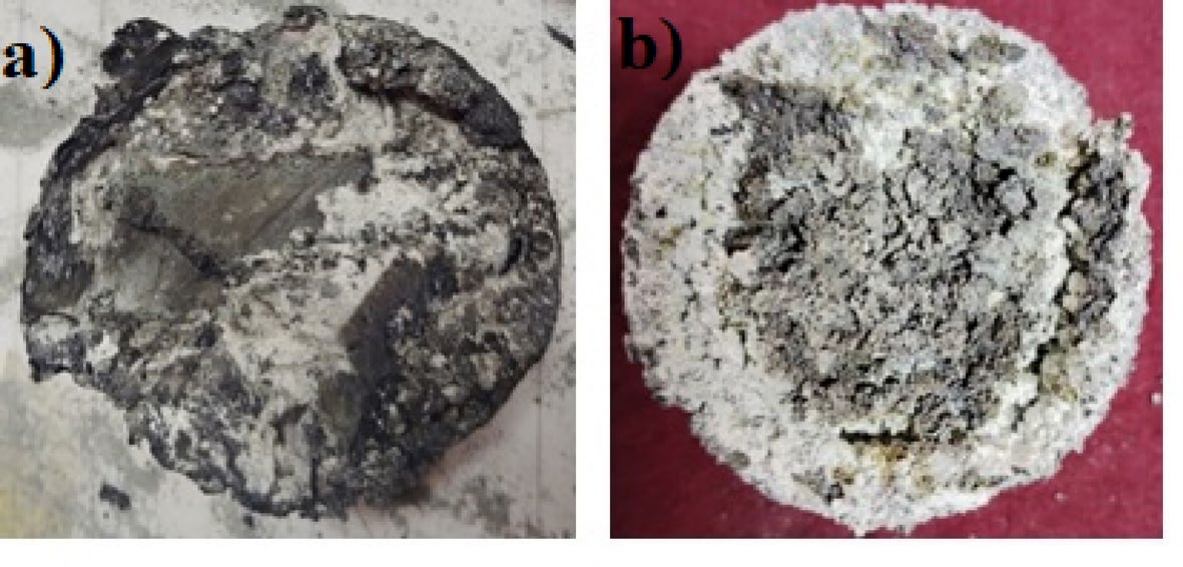
Images of the heating residues formed on top of AZ31 Mg alloy; (**a**) Exp-6: 550 °C with argon gas shielding in the Resistance furnace, (**b**) Exp-8: 630 °C with argon gas shielding in the Resistance furnace.

Figure 6 illustrates the typical surface appearance of molten Mg AZ31 alloy in Exp-6 and Exp-8, showing nodular residues forming on the granules in the resistance furnace. However, in the muffle furnace under the same conditions, the material did not melt. In the absence of argon protection, all samples completely burned in both furnaces. The elevated heating temperatures caused rapid oxidation of the granules, resulting in a substantial build-up of MgO on their surfaces, as shown in Fig. 6. As the temperature increases, the evaporation rate of Mg intensifies, allowing it to infiltrate the cracks and pores within the oxide layer. Upon contact with oxygen, the MgO leads to the formation of nodules that coalesce. Consequently, a continuous but heterogeneous oxide layer with granular, cauliflower morphology was exhibited on the surface^30,36^. This phenomenon is significantly enhanced with longer heating durations. Examining Fig. 6**(a)** reveals that after heating at 550 °C, a substantial amount of black heterogeneous nodular residue formed on the surface of the specimen. Figure 6**(b)** shows that upon heating to 630 °C, the residue transitions to relatively greyish nodules. As the heating temperature increases, a greyish residue appears more uniformly across the granules as depicted in Fig. 6**(b).** The appearance of a grey residue signifies extensive burning of the granules throughout the heating process. Furthermore, the transition of the residue colour from black to grey with rising heating temperatures implies that the black residue produced at lower temperatures probably contains compounds besides MgO, since pure magnesium oxide is grey. The higher heating temperatures intensify the burning of the Mg alloy at the top portion of the specimen, leading to vigorous vapour generation beneath the residue, which may not escape completely. Consequently, the development of pressurised vapour causes openings in the residue formed on top of the granules, as illustrated in Fig. 6**(b).**

### Microscopic observations

**Fig. 7:**
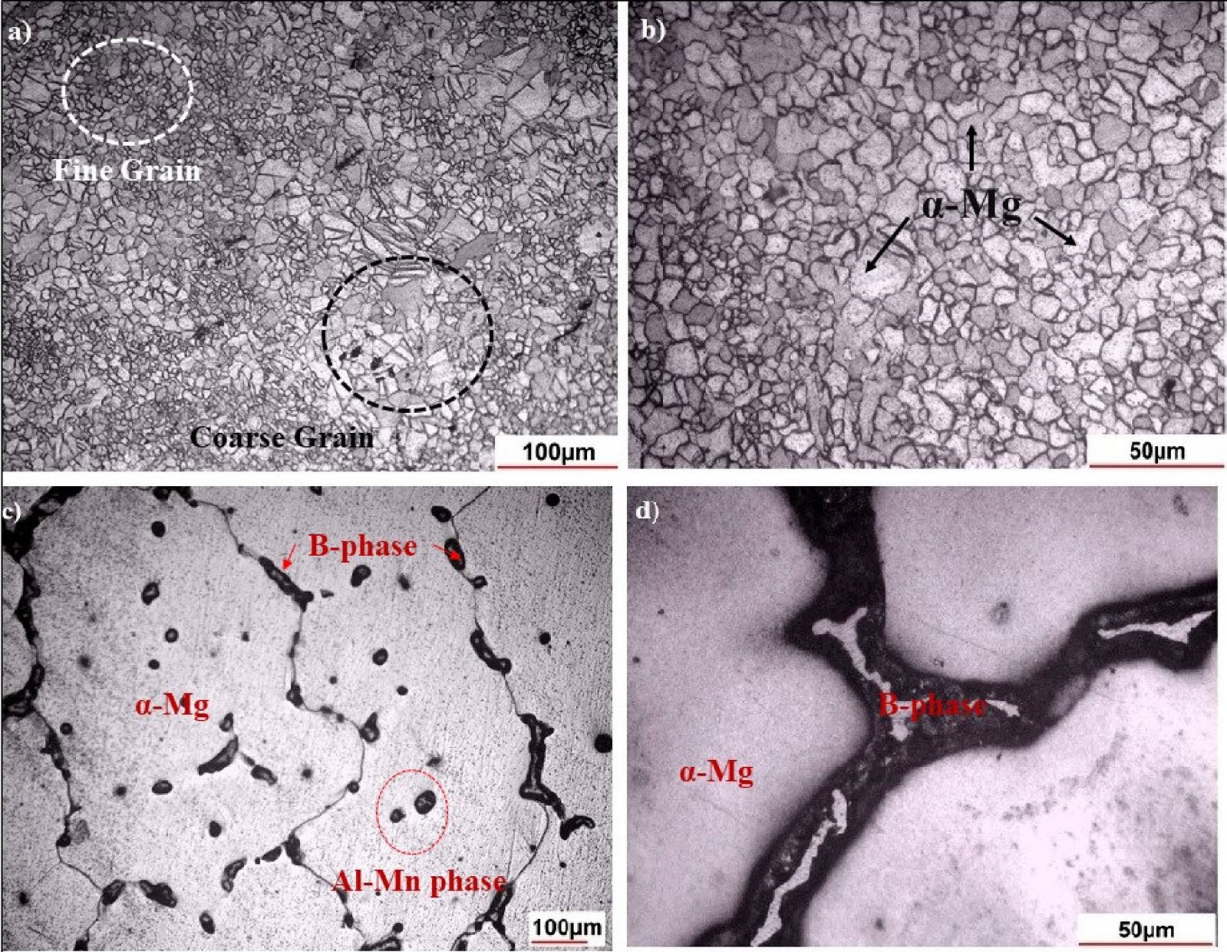
Optical micrographs of AZ31 Mg alloy: (**a**, **b**) As-received Rolled sheet; (**c**, **d**) Cast sample heated at 630 °C under argon in a resistance furnace.

**Fig. 8:**
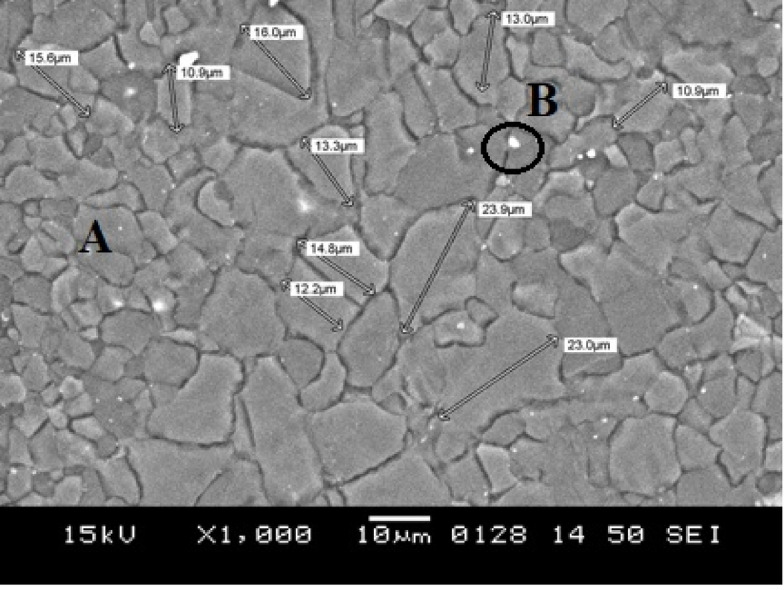
SEM Micrograph of as-received roll sheet (raw material) AZ31 Mg alloy.

**Fig. 9:**
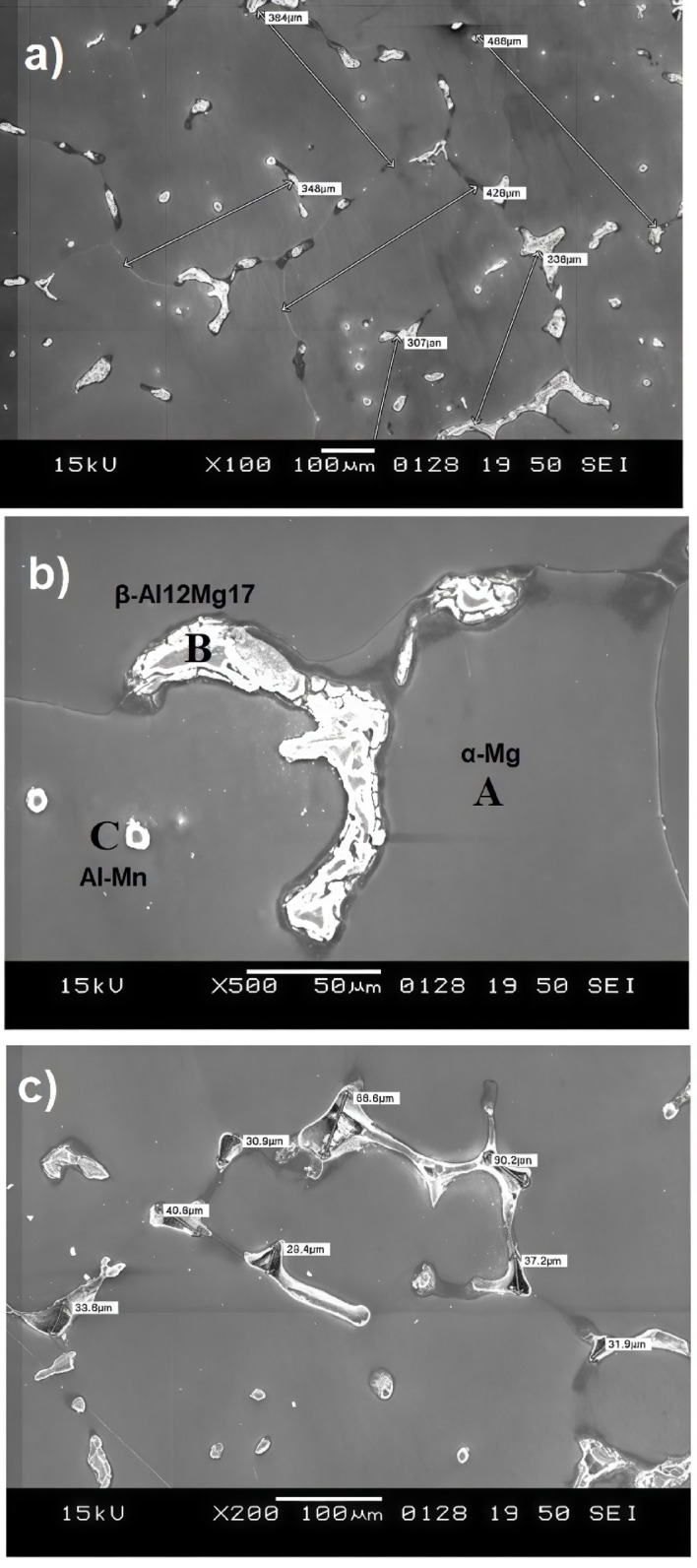
SEM micrographs of cast AZ31 Mg alloy: (**a**) Overall morphology showing distributed intermetallic networks; (**b**) Identification of α-Mg, β-Mg₁₇Al₁₂, and Al–Mn phases; (**c**) Pores and voids formed during solidification.

The surface film morphologies of the raw material and successfully cast (in Exp-8) Mg AZ31 alloy were evaluated. Imaging methods, including optical microscopy (OM) **(**Fig. 7**)** and SEM **(**Figs. 8 and 9**)**, were used for this analysis. Surface morphologies under other conditions were not captured due to improper melting and instances of burning. To analyse the oxidation behaviour of the AZ31 Mg alloy, SEM and OM were utilized to observe changes in surface morphology, composition, and microstructure following exposure to oxidizing conditions. After oxidation, the surfaces of the cast Exp-8 sample were analysed using SEM and compared with the as-received roll sheet (raw material) to identify any changes caused by the process.

The microstructure of the as-received AZ31 Mg alloy roll sheet (raw material) is shown in Fig. 7(a, b). The alloy exhibits a partially annealed structure with a clear bimodal grain size distribution, a common feature in rolled AZ31 alloys. In Fig. 7(a), both fine and coarse α-Mg grains are distinctly visible, with fine grains concentrated in localized zones and coarse grains appearing more irregularly distributed. This bimodal structure is further confirmed by SEM analysis (Fig. 8), which reveals smaller grains in the range of 10–13 μm and larger grains between 15 and 24 μm. The presence of such a grain distribution can enhance the mechanical properties of the alloy by offering a balance of high strength (due to fine grains) and improved ductility (due to coarse grains)^37,38^. The microstructure consists of small, irregularly shaped grains with relatively straight surfaces. The grains mostly appear equiaxed, meaning they are roughly equal in all dimensions, with new grains forming with slight deformation. The irregular shapes may suggest mechanical deformation or significant anisotropy in the material properties^39^. The image exhibits a grainy texture with clearly visible boundaries, which is characteristic of a polycrystalline material. The dark lines indicate grain boundaries, separating individual grains in the alloy. There is no evidence of twinning within the grains, and the microstructure appears inhomogeneous. The grain boundaries and structure significantly influence the alloy’s mechanical properties, with finer grains generally improving strength and hardness, as explained by the Hall-Petch relationship^40,41^.

The globular-dendritic microstructure of the as-cast alloy is significantly different from that of the raw material, as depicted in Fig. 7**(c**,** d)**. The cast AZ31 alloy structures an inhomogeneous grain size distribution with notably large grains, as shown in the figure. The grain sizes in Fig. 9**(a)** range from 307 μm to 488 μm, significantly more extensive than those observed in the SEM image of the raw material. This heterogeneity in grain size is typical of cast materials. Variations in cooling rates across different regions during casting can result in a non-uniform grain structure, causing some grains to grow more significantly than others, and grain coarsening occurs^42,43^. The figure shows that the cast AZ31 Mg alloy primarily comprises grains and a substantial number of small second-phase particles. The precipitated phase exhibits an irregular distribution within the alloy. During the heat conservation process, the number of grains decreases as the temperature increases, while the average grain size increases, resulting in grain growth. At higher temperatures, more heat is absorbed, storing significant energy within the grains. This causes an increase in grain boundary mobility, and the small second-phase particles merge into the larger grains, leading to a more uniform grain distribution and further grain growth^44^. It is well known that AZ31 Mg alloys primarily contain the α-Mg phase and the secondary β-Al_12_Mg_17_ phase. The primary α-Mg phase is the Mg-rich matrix phase in the AZ31 alloy. This phase forms the bulk of the Mg alloy and appears as the darker, continuous background in Fig. 9**(b).** This phase is the smooth, relatively featureless region surrounding the brighter, more complex shapes. It is generally softer and more ductile, contributing to the overall mechanical properties of the alloy. The colourful, irregularly shaped regions in the image represent the β-Mg₁₇Al₁₂ phase, an intermetallic compound that forms in the AZ31 alloy, particularly during solidification and subsequent cooling. It typically precipitates at grain boundaries or interdendritic regions with high aluminum (Al) and Mg concentrations. This phase often forms in a eutectic structure or as discrete precipitates, significantly affecting mechanical properties such as hardness and brittleness. The relatively sizeable interdendritic spacing in the SEM image is consistent with a slow cooling rate during casting, contributing to a coarser microstructure. The Al-Mn phase appears as particles containing Al and manganese (Mn), often discrete particles within the Mg matrix. These particles can act as nucleation sites for the precipitation of other phases, thereby enhancing the alloy’s overall mechanical properties and corrosion resistance. The absence of twins in this figure suggests that deformation twins are not present in the as-cast condition, which may seem intuitive^45^. Because, during casting, the rapid cooling and lack of significant plastic deformation typically prevent the formation of twins, which are more likely to form under specific mechanical stresses during processes like rolling. The dark, irregular areas in Fig. 9**(c)** represent pores formed during the solidification of the AZ31 Mg alloy, often caused by trapped gases or cooling shrinkage. SEM images show that after heating to 630 °C, lighter, brighter areas around the pores suggest an oxide layer formation. The thickness of the oxide layer increases when argon gas shielding is insufficient, allowing oxygen to interact with the molten metal. Prolonged heating results in the formation of additional MgO on the surface, which thickens the oxide layer due to the penetration of oxygen through the loose, non-protective oxide. Mg ions and vapor also diffuse through cracks and pores, reacting with oxygen and causing cracks between the oxide layer and the substrate during grinding or polishing due to MgO’s loose structure^30,46^. Rapid evaporation of Mg creates more voids, forming pathways for Mg vapor transportation, which generates local stresses and cracks^47^. These cracks fill with Mg vapor, which condenses and oxidizes, further increasing oxide thickness. This process shows selective oxidation along grain boundaries, particularly of the molten β-Mg_17_Al_12_ phase, which seeps through the oxide layer and oxidizes, increasing oxide thickness. After cooling, the unoxidized β-Mg_17_Al_12_ phase solidifies at grain boundaries. Even with an oxide layer, oxygen penetrates to react with the substrate, expanding evaporation areas and oxide volume. Observations indicate an increasing oxide formation trend when the alloy is heated from 550 °C to 630 °C. Beyond this temperature, voids and cracks allow oxygen penetration, promoting further oxidation and dramatically increasing oxide formation. The coarse-grain structure in cast AZ31, though reducing strength, may enhance corrosion resistance and promote controlled degradation features desirable for temporary biodegradable implants through cost-effective casting methods^48–50^.

### XRF, EDS, and XRD analyses

The XRF analysis results for the as-received rolled sheet and cast Exp-8 sample AZ31 Mg alloy are provided in Table 1. A comparison of the data shows slight variations attributed to the casting process. The observed differences between the rolled and cast compositions are expected due to thermal and metallurgical changes occurring during the casting process. The cast sample exhibits a slightly higher Mg concentration, possibly due to the evaporation or oxidation of other elements during casting. This leads to a higher concentration of Mg in the final product. Mn content also shows a slight increase in the cast alloy, suggesting that casting might cause some segregation of Mn into the matrix, potentially improving the alloy’s corrosion resistance and mechanical properties^51^. In contrast, Al and zinc (Zn) levels are lower in the cast AZ31 alloy. The Al reduction is likely due to its migration to the grain boundaries during solidification, which lowers its concentration in the overall matrix. With its lower melting point, Zn may have evaporated or segregated during casting at the high temperatures involved. This decrease in Al and Zn could reduce strength and hardness, potentially making the cast alloy less robust than the roll sheet.

In comparing the XRF results for the Roll Sheet AZ31 and Cast AZ31 Mg alloy samples, the differences in oxide content due to casting methods can be observed, which subsequently affect mechanical properties and structural phases. For the Roll Sheet AZ31, MgO dominates at 96.010%, with noticeable amounts of Al₂O₃ (2.939%) and minor components like SiO₂, MnO, and ZnO. In contrast, the Cast AZ31 has slightly more MgO (96.623%) but exhibits higher impurity levels in elements such as SO₃, Cl, K₂O, and CaO, whiExpch are relatively higher than in the roll sheet. For instance, Cl content in Cast AZ31 is 818.7 ppm compared to 429.6 ppm in the Roll Sheet. The increased impurity content in the Cast AZ31 can lead to microstructural defects, such as porosity, that weaken the mechanical properties, making it more prone to brittleness. Higher oxide inclusions, especially MnO and Fe₂O₃ in the cast sample, could decrease hardness. On the other hand, the raw material is likely to have more homogeneous properties due to lower levels of these impurities, contributing to better mechanical behaviour, especially in malleability and fatigue resistance, and prohibiting the initiation of edge cracks^8,52^.

**Fig. 10:**
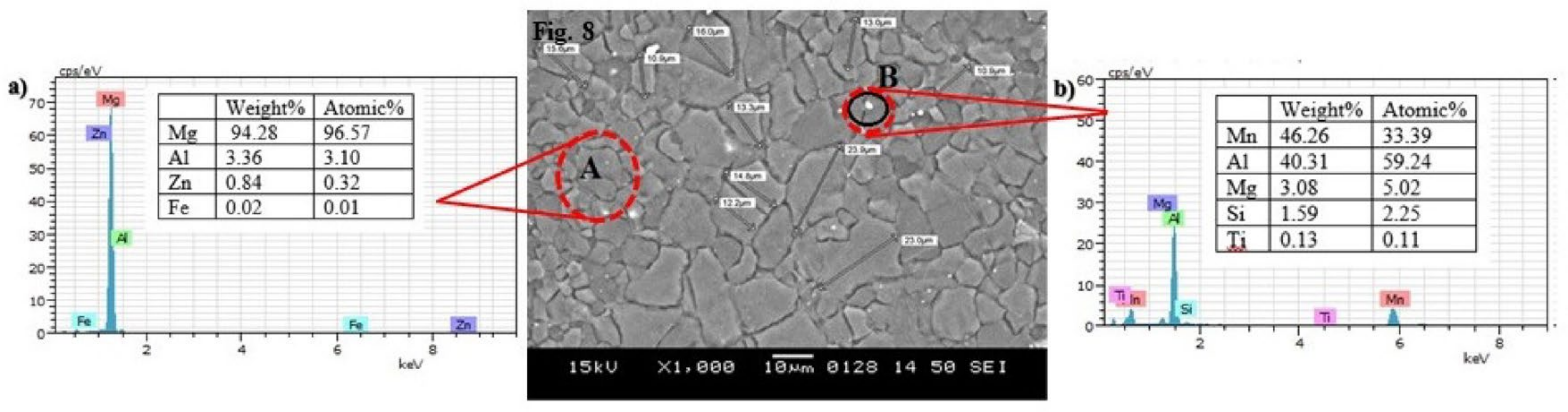
EDS spectra of as-received AZ31 Mg alloy roll sheet: (**a**) and (**b**) corresponding to the A and B positions shown in Fig. 8, respectively.

**Fig. 11:**
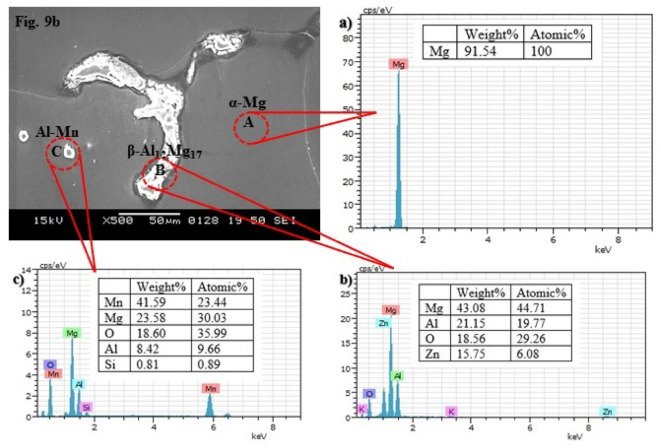
EDS spectra of Cast AZ31 Mg alloy: (**a**), (**b**), and (**c**) corresponding to the A, B, and C positions shown in Fig. 9(b), respectively.

The EDS analysis performed on the as-received roll sheet and cast AZ31 Mg alloy is illustrated in Figs. 10 and 11, respectively. In Fig. 10**(a)**, the high percentage of Mg by weight indicates that this area corresponds to the primary α-Mg phase, which constitutes most of the alloy. This high Mg content aligns with the SEM image, which depicts a relatively uniform distribution of the primary phase. Al, Zn, and Iron (Fe) are present in smaller quantities, likely dispersed throughout the Mg matrix and contributing to the formation of secondary phases. In Fig. 10**(b)**, Mn and Al are predominant in this region, with notable weight percentages. This indicates the presence of an Al-Mn intermetallic phase or localized segregation. These phases may appear as distinct features along the grain boundaries or as separate particles, adding to the microstructure’s heterogeneity. The presence of Silicon (Si) and Titanium (Ti), though in smaller quantities, suggests the existence of additional phases or inclusions within the alloy. These elements could form discrete particles or be incorporated into the matrix in specific areas.

The EDS spectrum in Fig. 11**(a)** reveals that the area predominantly comprises Mg, indicating that this region consists almost entirely of pure Mg, characteristic of the α-Mg phase in Mg alloys. The absence of an Al peak in the spectrum suggests that the oxide formed on the granules is primarily MgO, consistent with previously reported findings^36,53^. In Fig. 11**(b)**, the EDS spectrum shows a significant amount of Al, confirming the presence of the β-Mg_17_Al_12_ phase, a secondary phase that is rich in Al. The EDS spectrum in Fig. 11**(c)** displays a high concentration of Mn, along with Mg, O, Al, and a small amount of Si, suggesting the presence of a Mn-Al intermetallic compound in this region. EDS line scan microanalysis has confirmed that oxygen has penetrated the alloy’s oxide layer, resulting in oxidized products formation. The oxygen content increases in these areas while the Mg content decreases.

**Fig. 12:**
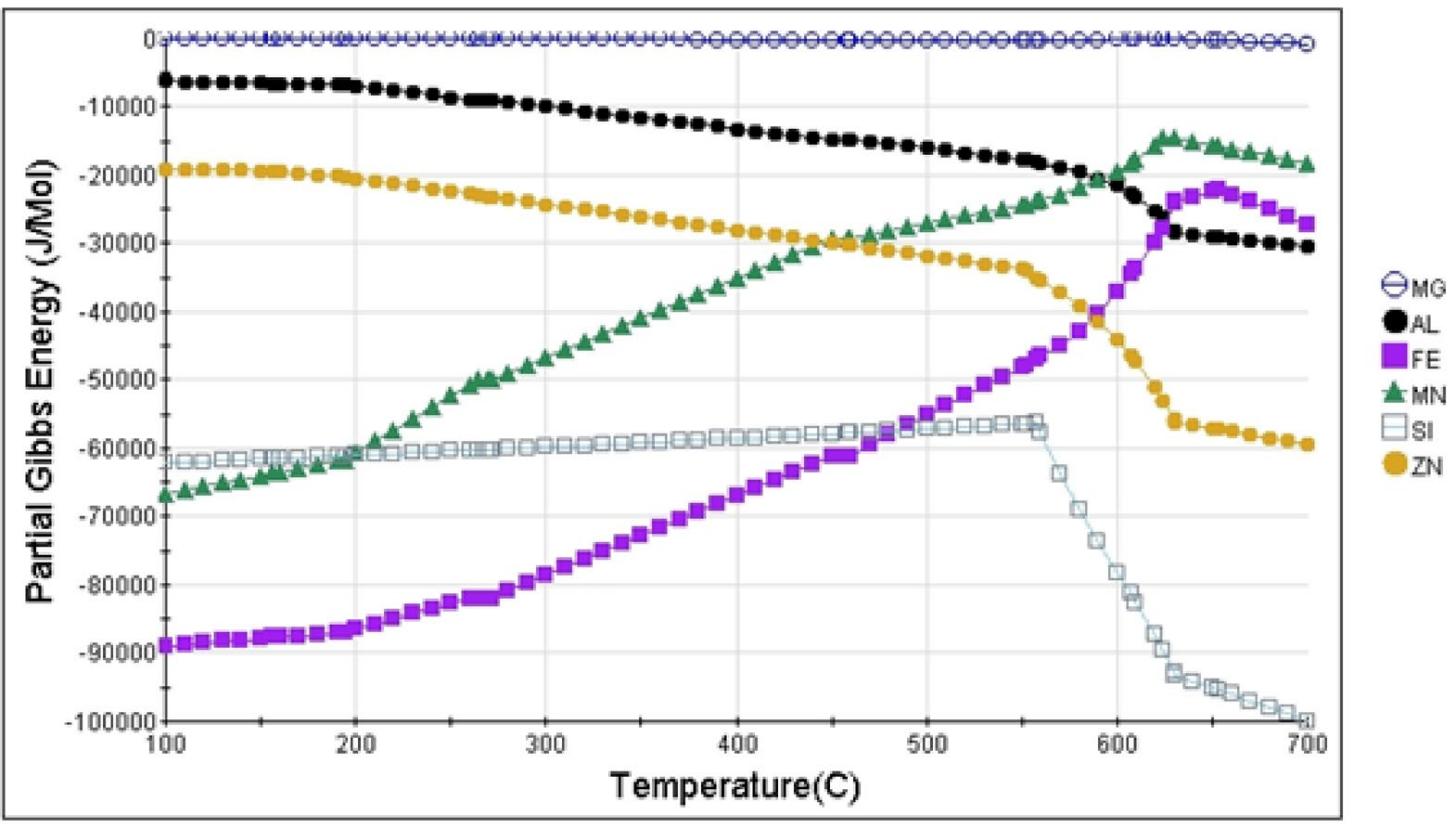
Effect of temperature on the change in Gibbs Standard free energy (calculated by JMatPro Version 7.0.0 software).

**Fig. 13:**
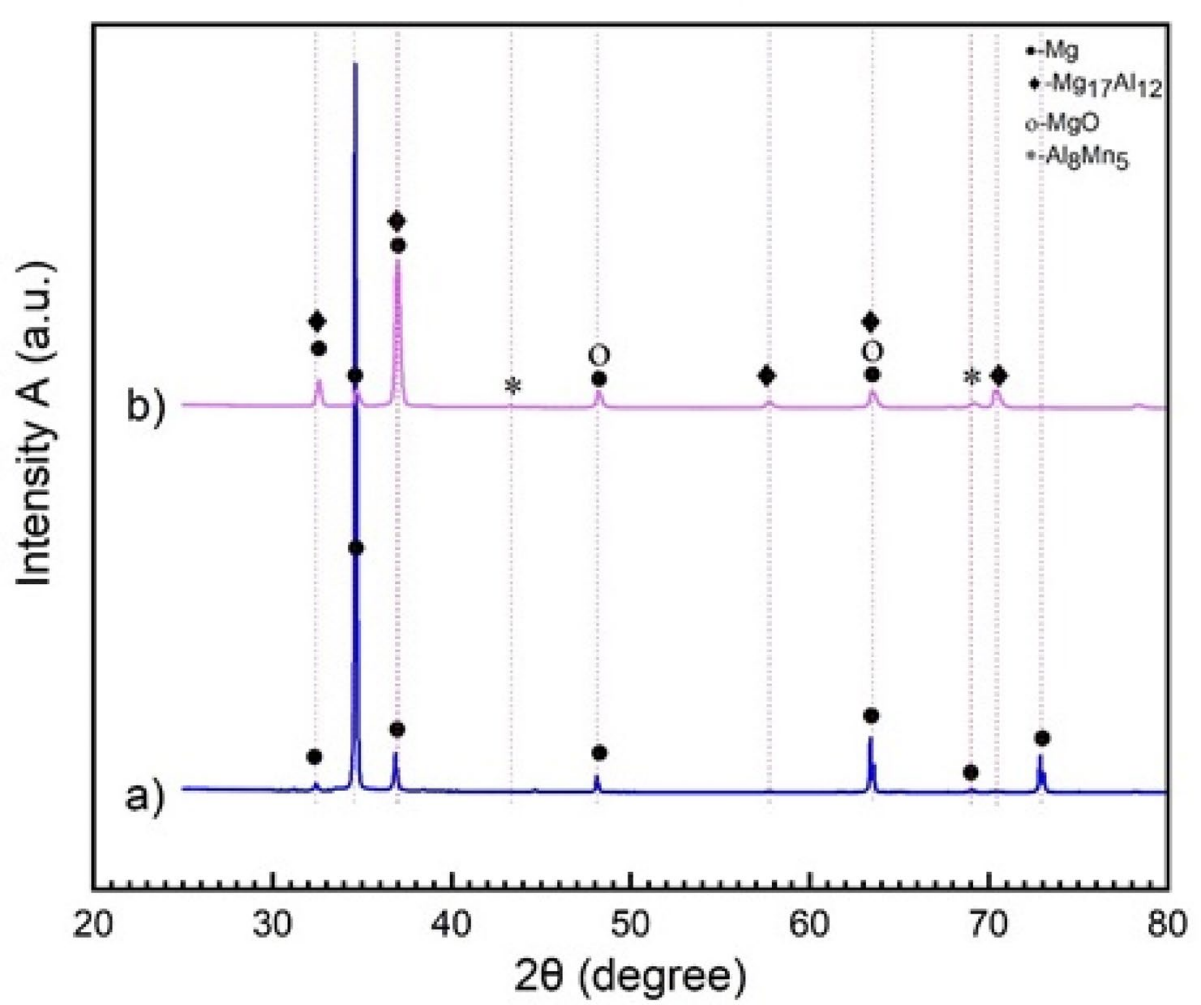
XRD graph for specimens (**a**) as-received roll sheet (raw material), (**b**) cast AZ31 Mg alloy.

The graph **(**Fig. 12**)** from the JMatPro (Version 7.0.0) software illustrates the partial Gibbs free energy of different elements in the AZ31 Mg alloy changes with temperature, indicating phase stability and possible phase transitions. The combined analysis of Gibbs free energy and XRD peaks helps to identify the formation and stability of various phases in the AZ31 Mg alloy. The thermodynamic data provide a predictive context for understanding which oxides or intermetallic phases will likely form under different temperature conditions. At the same time, the XRD results offer experimental validation of these predictions^54^, particularly in identifying the stable and metastable phases present in the alloy at various temperatures^55^. The Gibbs free energy (ΔG) measures how spontaneously a reaction occurs, with more negative values indicating greater spontaneity. In the case of AZ31, the analysis revealed that Mg exhibited stable behaviour across the entire temperature range, with minimal changes in Gibbs free energy. This stability suggests that pure Mg remains a dominant phase in the alloy, confirmed by firm XRD peaks corresponding to Mg. The role of Al is critical in the formation of intermetallic phases such as Mg_17_Al_12_. The Gibbs free energy curve for Al showed an increasing trend at elevated temperatures, indicating that the stability of Al-containing phases, such as Mg_17_Al_12_, is temperature-dependent. The XRD patterns **(in** Fig. 13**)** supported this finding, with clear peaks corresponding to Mg_17_Al_12_, a phase that forms due to the interaction between Mg and Al at intermediate temperatures. The alignment between the rise in Gibbs free energy for Al and the appearance of Mg_17_Al_12_ peaks highlights the ability of Gibbs energy calculations to predict phase evolution under thermal treatment. Similarly, the Gibbs energy values for other elements like Al, Fe, Mn, Si, and Zn indicate that while their oxidation is thermodynamically feasible, the extent of their tendency to oxidize varies. Al has a negative Gibbs energy, but not as negative as Mg, indicating that while it can form an oxide (Al₂O₃), its oxidation is less favoured than Mg. Al forms Al³⁺ ions with a slightly larger ionic radius and partially covalent character in Al₂O₃, making its oxidation less favourable than Mg formation of MgO. Mg more negative standard reduction potential means it oxidizes more readily than Al, reflecting a stronger tendency for Mg to form its oxide. This phase was detected in the XRD analysis, with distinct peaks corresponding to MgO, confirming the prediction made by the thermodynamic data. At higher temperatures, the Gibbs energy changes for elements like Mn, Si, and Zn suggest their oxides may form more readily. Mg’s Gibbs energy remains negative and relatively stable, indicating that its oxidation is still spontaneous at elevated temperatures. In contrast, Mn shows a more significant increase in Gibbs energy, suggesting that its oxidation might become less favourable. Despite this, Mn has a strong tendency to oxidize, especially at temperatures like 630 °C, where the formation of manganese oxides remains thermodynamically favorable. In an environment conducive to oxidation (with sufficient oxygen and temperature), Mn would form stable oxides preferentially, resulting in a higher concentration of Mn in the oxidized regions detectable by EDS. If other elements in the sample, such as Al or Si, have less negative Gibbs energies for their oxides at 630 °C, they would oxidize less readily than Mn. As a result, Mn would dominate in the oxide phases, leading to its higher detection in the EDS image. The XRD analysis corroborates this by detecting Mn-rich phases, such as Al_8_Mn_5_, which form under specific temperature conditions. The preferential formation of manganese oxides at high temperatures is likely due to the thermodynamic favourability indicated by Gibbs energy values. It can be further confirmed through EDS analysis, where Mn is detected more prominently in the oxidized regions of the sample.

X-ray diffraction (XRD) was performed to analyse the phase composition and oxide formation behaviour of AZ31 Mg alloy. The diffraction patterns revealed distinct peaks corresponding to α-Mg (JCPDS No. 35–0821), β-Mg₁₇Al₁₂ (JCPDS No. 01–1128), Al₈Mn₅ (JCPDS No. 49–1279), and MgO (JCPDS No. 79–0612). Phase identification was conducted using the X’Pert HighScore Plus (Version 3.0.0, by PANalytical B.V. Almelo, The Netherlands) software with an integrated crystallographic database. To evaluate the effect of casting conditions on phase evolution, eight experimental trials (Exp-1 to Exp-8) were conducted under different furnace types, temperatures, and atmospheric conditions. Due to excessive oxidation in Exp-1 to Exp-4 and incomplete melting in Exp-5 to Exp-7, only Exp-8 produced a fully cast sample. The XRD patterns from all eight trials are provided in Supplementary **Fig. S1.** For a detailed comparison, Fig. 13 presents the XRD spectra of the as-received AZ31 rolled sheet (raw material) and the successfully cast Exp-8 sample, which were further analysed to assess phase formation and microstructural transitions.

Figure 13(a) shows the XRD spectrum of the raw material, which consists of a sharp and narrow peak corresponding to the α-Mg phase, indicating a predominantly single-phase structure. This indicates that the rolling process, possibly followed by heat treatments, has produced a homogeneous microstructure where the α-phase predominates^56^. The α-phase, characterized by its hexagonal close-packed (HCP) crystal structure, is typical of Mg alloys. It forms the alloy’s matrix and is responsible for its mechanical properties. The lack of secondary intermetallic reflections in the XRD spectrum implies that these phases have either dissolved during rolling or are present in amounts below the detection threshold of the technique^57–59^. The absence of significant peaks corresponding to secondary phases such as the β-phase, MgO, or intermetallic compounds in the XRD pattern of the raw material suggests that the rolling process has minimized the formation of these phases. This outcome aligns with the effects of dynamic recrystallization during rolling, which refines the grain structure and can dissolve or more homogenously distribute secondary phases throughout the matrix with minimal phase segregation or residual stress^58–61^. These findings are consistent with the OM in Fig. 7(a, b), which reveals a bimodal grain structure composed of fine and coarse equiaxed α-Mg grains with a typical feature of continuous dynamic recrystallization (CDRX) during thermomechanical processing. Such refined structures contribute to improved uniformity and enhanced mechanical performance^58,60^. The discrepancy between EDS and XRD results highlights the complementary nature of these techniques. EDS reveals localized compositional details and can detect small or dispersed secondary phases, while XRD provides a bulk-phase composition and identifies predominant crystalline phases. The observed contradiction can be explained by the presence of secondary phases or inclusions that are localized and detected by EDS but not in sufficient quantities to alter the XRD pattern significantly. The rolling process likely refined the primary α-Mg and dispersed secondary phases, resulting in a homogeneous bulk phase composition that XRD can detect. At the same time, EDS captures localized compositional variations^62^. The lack of these secondary phases in the rolled sheet likely enhances the material’s ductility and toughness, as the brittle β-phase and other intermetallic compounds can serve as crack initiation sites, possibly compromising the material’s overall performance^63^.

In Fig. 13**(b)**, like the raw material, the α-phase (Mg) is present and remains the dominant phase. The prominent peaks in the spectrum correspond to the α-phase (Mg), the primary solid solution phase in the AZ31 alloy. The α-Mg phase was obtained at different intensities with different lattice parameters. This phase is characterized by a hexagonal close-packed (HCP) structure. This phase forms the alloy’s matrix, providing the base for its mechanical properties. In the context of casting, the α-phase may be coarser due to slower cooling rates, resulting in larger grain sizes. The β phase appears as secondary peaks in the XRD pattern. This intermetallic compound has a body-centred cubic (BCC) structure and typically forms in Mg alloys when the Al content is sufficient. The formation of the β phase is influenced by processing conditions, particularly temperature and cooling rate^64^. It typically forms along grain boundaries (Fig. 9) during solidification in cast alloys. The β-phase is known for being hard and brittle, which can increase the alloy’s strength but decrease its ductility. Its presence in cast AZ31 is common, especially when Al segregates during solidification. MgO forms due to the oxidation of Mg, which can occur during the casting process, even with argon shielding. This phase is generally not desirable as it can lead to inclusions that act as stress concentrators, potentially reducing the mechanical properties of the alloy. In an Al-containing Mg alloy, Mn can form the Al–Mn phase in the microstructures. The Al–Mn phases are mainly Al_4_Mn, Al_6_Mn, Al_8_Mn_5,_ and Al_11_Mn_4_^65^ Using EDS, it was determined that the atomic Al/Mn ratio was close to 1:2.44. Hence, combined with the morphology of the Al-Mn phase and from Fig. 4, they were most likely Al_8_Mn_5_^66,67^. The Al₈Mn₅ intermetallic phase forms due to the interaction between Al and Mn, and plays a crucial role in determining the microstructure during the solidification of the cast AZ31 Mg alloy^66^. It usually forms as small, discrete particles within the microstructure and can act as nucleation sites during solidification^68^. As shown in the Al–Mn binary phase diagram (Fig. 4), Al₈Mn₅ precipitates from the melt at approximately 630 °C, before the formation of primary α-Mg and β-Mg₁₇Al₁₂ phases^66^. At the end of solidification, the remaining liquid transforms into α-Mg and β-Mg₁₇Al₁₂, resulting in Al₈Mn₅ particles being bound by bright central regions of the solid phase, as observed in Fig. 9.

Due to its large lattice mismatch with α-Mg, Al₈Mn₅ is expelled to grain boundaries, whereas its structural compatibility with β-Mg₁₇Al₁₂ allows it to act as a favourable nucleation substrate for the eutectic phase^69^. When finely and uniformly dispersed, Al₈Mn₅ contributes to grain boundary pinning, restricts grain coarsening, and promotes a more equiaxed grain morphology. In contrast, clustering of Al₈Mn₅ in solute-rich regions may encourage β-phase coarsening and lead to precipitation-free zones (PFZs), reducing microstructural uniformity. Furthermore, these clusters, particularly when associated with shrinkage porosity or interdendritic voids, can act as stress concentrators, promoting localized cracking and embrittlement^70^. Thus, the effectiveness of Al₈Mn₅ as a grain refiner and stabilizing phase is highly dependent on its size, distribution, and interaction with the evolving solidification front. A well-dispersed Al₈Mn₅ phase supports microstructural refinement, whereas excessive clustering can compromise mechanical performance. Additionally, the XRD patterns reveal that no zinc oxide was detected. This absence is attributed to zinc oxide being less stable than Mg and Al at the temperatures studied. Zn does not actively participate in the oxidation process, but its presence in the alloy lowers the ignition temperature of Mg. This effect is likely due to Zn’s role in promoting the formation of the low-melting Mg_17_Al_12_ phase within the alloy’s microstructure, increasing the oxidation rate. During heating, the Mg_17_Al_12_ phase melts easily, whether in a continuous or discontinuous form. This melting accelerates the oxidation process due to the presence of the liquid phase. Furthermore, the liquid phase can disrupt the oxide layer, leading to increased evaporation of the Mg alloy, with Zn often evaporating alongside Mg. The lack of zinc oxide in the XRD patterns, even though it was identified in the EDS analysis, indicates that Zn could have evaporated alongside Mg during the heating process. Some Zn may have condensed or been trapped within voids or pores. Nevertheless, as mentioned, the quantity of Zn present in the oxidation product is minimal^30,71^. The broader peaks in the XRD pattern of the cast sample reflect both larger grain sizes and the presence of multiple phases, indicative of a more heterogeneous microstructure compared to the raw material. In comparison, the cast sample (Fig. 7c, d) reveals non-uniform grain morphology, along with visible porosity and second-phase particle clusters, which are typical of solidification structures in unmodified AZ-series alloys^72^. While the presence of hard intermetallic phases like β-Mg₁₇Al₁₂ and MgO may improve hardness and strength, they often reduce ductility and toughness, properties that are critical for formability in structural applications. The microstructure is more heterogeneous, with anisotropic properties arising due to possible phase segregation.

### Mechanical properties

The mechanical properties of AZ31 Mg alloy, particularly density, porosity, and hardness, exhibit substantial variation based on the manufacturing method. Tests were conducted on the raw material and the successfully cast sample obtained from Experiment 8 (resistance furnace, 630 °C, with argon shielding). This was the only trial that produced a casting specimen suitable for mechanical testing, whereas the other experiments were severely oxidised or incomplete. The results revealed marked differences in structural integrity and mechanical performance between the rolled and cast conditions. In each experiment, specimens were produced with dimensions of 40 mm in diameter and 2 mm in width.

The raw material was heavier than the cast sample despite being fabricated from the same AZ31 Mg alloy. This discrepancy is directly related to the size and volume fraction of porosity present in the cast sample. With its denser and nearly pore-free structure, the raw material exhibits a greater mass for the same volume than the cast sample. In contrast, the cast sample contains larger, irregular pores resulting from gas entrapment and shrinkage during solidification, significantly lowering its weight. The overall mass is reduced due to the diminished material volume caused by the high porosity. The absence of significant porosity in the raw material results in a compact, solid structure capable of bearing greater loads, contributing to its higher weight and superior mechanical strength. In contrast, the cast sample, with pores averaging 40 μm in size in Fig. 9**(c)**, suffers from reduced strength and load-bearing capacity due to the weakening effects of these voids^73^.

The density measurements were obtained using Archimedes’ principle^74^. The theoretical density was calculated by dividing the mass of each sample by its volume, providing an average theoretical density for comparison. The raw material displayed a measured density of 1.776 g/cc, which is close to the theoretical density of AZ31 Mg alloy^20^. This suggests a compact, defect-free structure with minimal porosity from the compressive forces involved in the rolling process, ensuring a uniform and dense microstructure. In contrast, the cast sample had a significantly lower density of 1.487 g/cc, which can be attributed to the internal porosity introduced during the casting process. The voids and shrinkage defects generated during solidification, particularly from gas entrapment, contribute to this reduction in density.

Porosity, expressed as a percentage ranging from 0% to 100%, was calculated by comparing the measured and theoretical densities using the following Eqs^74,75^. :

*% porosity = (1-(Measured density/theoretical density)) X100*.

The raw material, with 1.69% porosity, indicates a high-quality microstructure with minimum voids. During rolling, the material undergoes deformation and recrystallization, which refines the grain structure and minimizes internal flaws. Consequently, the rolled sheet exhibits superior mechanical properties, such as higher strength, hardness, and fatigue resistance, as the absence of pores eliminates stress concentrators. On the other hand, the cast sample, with a porosity of 16.4%, shows a considerable reduction in density and mechanical properties^73^. Pores, particularly large ones, degrade the material performance by acting as stress concentrators, making the cast sample more susceptible to crack initiation and failure under mechanical loading. The stirring conditions, pouring technique, and neutralization of the casting environment are the casting parameters that may be changed to effectively manage porosity, even if they cannot all be removed^76^.

**Table 3:** Mechanical properties comparison between the rolled sheet and cast AZ31 Mg alloy samples.

Property	Rolled sheet (AZ31)	Cast AZ31 sample	Change (Cast vs. Rolled)
Weight (g)	2.466	2.250	−8.75%
Density (g/cc)	1.740	1.487	−14.51%
Porosity (%)	1.69	16.40	+ 14.71%
Vickers Hardness (HV)	65.15	54.20	−16.83%

**Fig. 14:**
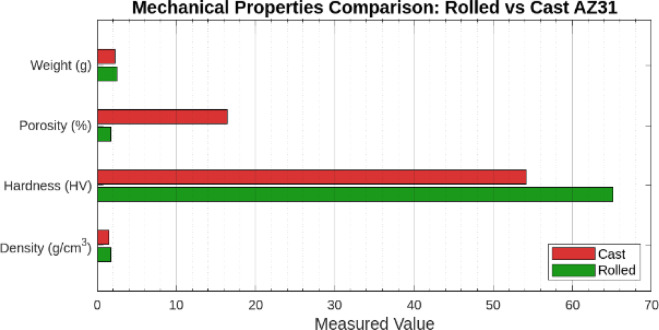
Comparison of mechanical properties: rolled vs. Cast AZ31 alloy.

The microhardness of the samples was measured using a Vickers microhardness testing machine equipped with a diamond indenter; the hardness test for samples was conducted according to ASTM E384-17 with the cylindrical shape of sample preparation under ASTM E3-95. In this study, a 50-gf load was applied with a dwell time of 10s. Five replicate measurements were carried out, and the average value was reported as microhardness^77^. The raw material average Hardness value is 65.15 HV, while the cast sample exhibited an average hardness of 54.2 HV. When comparing the hardness of the raw material to the cast sample, there is a noticeable percentage difference in hardness. The cast sample has approximately 16.83% lower hardness than the raw material. This reduction in hardness can be attributed to several factors closely linked to the microstructural and mechanical differences between raw and cast materials. The rolling process introduces work hardening into the sheet material, which increases its dislocation density, contributing to higher hardness. In contrast, cast materials typically have higher porosity and a coarser grain structure, which can reduce hardness^78^. These findings are summarized in Table 3 and visualized in Fig. 14, emphasizing the deterioration of mechanical properties in the cast AZ31 sample compared to the rolled sheet.

The formation of larger grain sizes in the cast material results from slower cooling rates during solidification, which limits the grain boundaries that can impede dislocation movement. Therefore, with the cooling rate, the mechanical properties of the alloy will deteriorate. The yield strength of the alloy is closely related to the microstructure, and the relationship between yield strength and average grain size satisfies the Hall-Petch relationship^79,80^.

3 $$\:{\sigma\:}_{y}={\sigma\:}_{o}+k{d}^{-1/2}$$

where *k* is the Hall-Petch slope, *d* is the average grain size, and *σ*_*0*_ is the intercept stress.

The rolled sheet, which undergoes substantial plastic deformation during processing, exhibits refined, equiaxed grains due to dynamic recrystallization (Fig. 7a, b). These fine grains act as effective barriers to dislocation motion, resulting in higher hardness and strength. In contrast, the cast sample, formed via slow cooling, retains coarser grains with dendritic features (Fig. 7c, d), which reduces grain boundary strengthening and results in a lower hardness value. Additionally, solid solution strengthening and the reduced presence of coarse β-Mg₁₇Al₁₂ intermetallic in the rolled sheet further enhance its mechanical properties. Grain coarsening, porosity, and discontinuous β-phase distribution in the cast sample promote stress concentration and crack initiation, weakening the overall structure^79,81^. This behaviour aligns with previous studies confirming the applicability of the Hall–Petch relationship across various Mg alloy processing routes^80–82^.

The cast AZ31 sample comprises a multiphase microstructure consisting of the primary α-Mg matrix along with intermetallic compounds such as β-Mg₁₇Al₁₂ and Al–Mn phases. Although these secondary phases can locally enhance hardness, they adversely impact the overall structural homogeneity and compromise mechanical performance. In particular, the β-Mg₁₇Al₁₂ phase, commonly precipitated at grain boundaries during solidification, is brittle and prone to crack initiation under mechanical loading, thereby reducing the material’s toughness and ductility^82,83^. The SEM micrographs (Fig. 9b) clearly show the presence of β-phase as intergranular networks, which interrupt the continuity of the α-Mg matrix and degrade its load-bearing capability. The reduced hardness and density observed in the cast sample can be further attributed to this phase. Although β-Mg₁₇Al₁₂ is intrinsically hard, its coarse and discontinuous morphology leads to mechanical mismatch at the grain boundaries. The eutectic reaction near the end of solidification transforms the residual melt into α-Mg and β-Mg₁₇Al₁₂, forming sharp interfacial boundaries that facilitate decohesion under stress, particularly in tensile conditions^84^. These regions become preferential paths for crack propagation due to their brittle nature and weak bonding with the matrix. In contrast, the rolled AZ31 sheet shows a homogeneous α-Mg matrix with minimal or no detectable β-phase. The rolling process, accompanied by dynamic recrystallization, refines the grain structure and suppresses the formation of coarse second phases. This microstructural uniformity translates into superior mechanical properties, including increased hardness, density, and structural integrity. The presence of porosity in the cast structure further exacerbates its mechanical limitations. As evident from the density and porosity data (Table 3), the cast sample contains shrinkage-induced voids and gas entrapment, acting as stress concentrators that accelerate failure under load. This microstructural deficiency is compounded by the presence of β-Mg₁₇Al₁₂, further reducing mechanical strength and hardness relative to the rolled counterpart^85^. Supporting evidence for the formation of these detrimental intermetallic is found in the XRD pattern of the cast sample (Fig. 13b), where distinct peaks for β-Mg₁₇Al₁₂ are observed. These peaks are notably absent in the XRD spectrum of the rolled sheet (Fig. 13a), confirming a more single-phase, α-Mg-dominant structure in the rolled condition. The cast sample’s slower cooling rate during solidification promotes solute segregation and facilitates the development of coarse secondary phases, thereby contributing to inferior mechanical performance compared to the rolled material^79,82^.

## Conclusions

This study highlights the significant impact of furnace type, temperature, and argon gas shielding on the oxidation behaviour and mechanical properties of AZ31 magnesium alloy during casting. The main findings can be summarised as follows.

1. The experimental results revealed that argon gas shielding effectively reduces oxidation but hinders heat transfer, leading to incomplete melting at 630 °C temperatures in the closed chamber furnace. While the open chamber furnace with argon shielding allowed for successful casting at 630 °C with minimal oxidation, the mechanical properties of the alloy deteriorated due to microstructural changes, such as grain coarsening and phase transformations.
2. Microscopic analysis revealed a coarse, heterogeneous grain structure in the cast samples due to uneven cooling, contrasted with the raw material had a finer, bimodal grain distribution. The presence of β-Al_12_Mg_17_ phase and oxide inclusions, especially in the cast alloy, negatively impacted mechanical performance.
3. XRF and EDS reveal significant changes in elemental composition and microstructure due to casting. The cast alloy exhibited a slight increase in Mg and Mn content but decreased Al and Zn levels, likely due to evaporation and segregation during casting. These compositional shifts resulted in reduced hardness in the cast alloy.
4. The XRD analysis, along with Gibbs free energy calculations, confirmed the formation and stability of key phases in the AZ31 alloy, including α-Mg, Mg₁₇Al₁₂, Al₈Mn₅, and MgO, thereby validating phase transitions under casting conditions. While the presence of multiple phases, such as the β-phase and MgO inclusions, could have contributed to locally high hardness in the alloy, their overall impact on the material behaviour was negligible compared to the influence of porosity.
5. The coarse-grain structure in the cast sample led to 14.71% higher porosity and 16.83% lower hardness than the raw material, reducing mechanical performance. However, this structure may enable controlled degradation in biodegradable implants, which requires confirmation through dedicated corrosion testing.

## Supplementary Information

Below is the link to the electronic supplementary material.

Supplementary Material 1

## Data Availability

The datasets used and/or analysed during the current study are available from the corresponding author (Gunvanta Dhanuskar at [gunvant.dhanuskar@gmail.com]) on reasonable request.
