# Supplementary material for: Investigating microstructural and mechanical properties evolution in biomedical AZ31 magnesium alloy under different casting conditions
Source: Sci Rep. 2025 Dec 29;15:44738. doi: 10.1038/s41598-025-28526-0 (PMC12748764; doi:10.1038/s41598-025-28526-0)
Supplement: Supplementary file 1 — Supplementary Material 1 [file 41598_2025_28526_MOESM1_ESM.docx]

***Supplementary Discussion***

***for***

**Investigating Microstructural and Mechanical Properties Evolution in Biomedical AZ31 Magnesium Alloy under Different Casting Conditions**

Gunvanta Dhanuskar **^a^**, Abhaykumar Kuthe **^a^,** Dheeraj Bhiogade **^a^**, Bhupesh Sarode **^a,^** and Ashutosh Bagde **^b,*^**

**^a^** Visvesvaraya National Institute of Technology, Nagpur, Maharashtra, 440010, India

**^b^** Datta Meghe Institute of Higher Education and Research, Wardha, Maharashtra, 442005, India.

**Supplementary Discussion**

**S1. Materials**

**Fig. S1** presents the X-ray diffraction (XRD) patterns of AZ31 magnesium alloy samples subjected to eight different experimental casting conditions (**Exp-1 to Exp-8**), highlighting the evolution of phase composition under varying furnace and atmospheric parameters. The observed diffraction peaks correspond to α-Mg, β-Mg₁₇Al₁₂, Al₈Mn₅, and MgO (oxidation product). In **Exp-1 to Exp-4**, the dominant presence of MgO and diminished α-Mg and β-Mg₁₇Al₁₂ peaks indicates severe oxidation due to uncontrolled environments, with residues likely originating from surface-oxidized or partially sublimated metal. **Exp-5 to Exp-7** show a gradual increase in β-Mg₁₇Al₁₂ and Al₈Mn₅ peak intensities, corresponding to partially melted samples with incomplete phase development, corroborating the macrographic evidence in **Fig. 5(e–g**). Notably, **Exp-8** reveals strong and distinct peaks of α-Mg, β-Mg₁₇Al₁₂, and Al₈Mn₅ with minimal MgO, indicating successful casting under argon-assisted resistance furnace conditions. This confirms complete solidification and proper phase formation in **Exp-8**, making it suitable for direct comparison with the rolled AZ31 sheet (raw material) in subsequent structural and mechanical analyses (**Fig. 13, Table 3**). The progression of phase formation across experiments underscores the importance of a protective atmosphere and thermal control during AZ31 casting in suppressing oxidation and promoting the desired intermetallic phase distribution, which is crucial for biomedical applications.


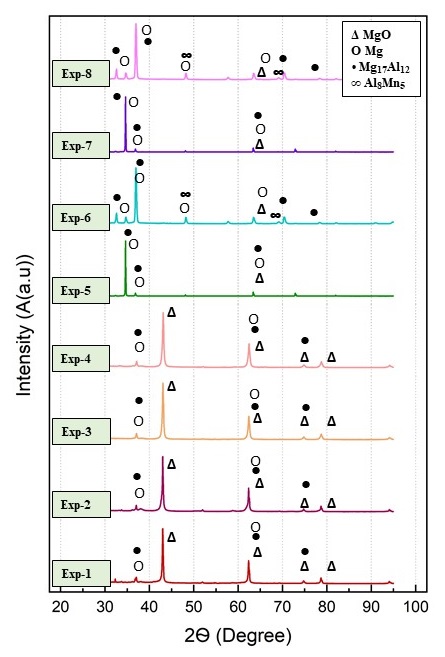


**Fig. S1.** XRD profiles of AZ31 magnesium alloy processed under eight experimental casting conditions (Exp-1 to Exp-8).
